# Overexpression of autophagy enhancer PACER/RUBCNL in neurons accelerates disease in the SOD1^G93A^ ALS mouse model

**DOI:** 10.1186/s40659-024-00567-1

**Published:** 2024-11-17

**Authors:** Luis Labrador, Leonardo Rodriguez, Sebastián Beltran, Fernanda Hernandez, Laura Gomez, Patricia Ojeda, Cristian Bergmann, Melissa Calegaro-Nassif, Bredford Kerr, Danilo B. Medinas, Patricio Manque, Ute Woehlbier

**Affiliations:** 1 Center for Integrative Biology, Faculty of Science, Universidad Mayor, Camino la Piramide 5750, P.O.BOX 70086, Huechuraba, Santiago Chile; 2 Escuela de Tecnología Médica, Facultad de Medicina y Ciencias de la Salud, Universidad Mayor, Camino la Piramide 5750, 8580745 Huechuraba, Santiago Chile; 3 Center for Biomedicine, Facultad de Medicina y Ciencias de la Salud, Universidad Mayor, Camino la Piramide 5750, 8580745 Huechuraba, Santiago Chile; 4 Biomedical Neuroscience Institute, Faculty of Medicine, University of Chile, Santiago, Chile; 5 Program of Cellular and Molecular Biology, Center for Molecular Studies of the Cell, Institute of Biomedical Sciences, University of Chile, Santiago, Chile; 6 Center for Geroscience, Brain Health and Metabolism, Santiago, Chile; 7 Centro de Estudios Científicos, Valdivia, Chile; 8 Centro de Biología Celular y Biomedicina (CEBICEM), Facultad de Medicina y Ciencia, Universidad San Sebastián, Lota 2465, 7510157 Santiago, Chile; 9 Department of Biochemistry, Institute of Chemistry, University of São Paulo, São Paulo, SP Brazil; 10 Centro de Oncología de Precisión (COP), Escuela de Medicina, Universidad Mayor, Santiago, Chile

**Keywords:** PACER, RUBCNL, KIAA0226L, Autophagy, Amyotrophic lateral sclerosis, Superoxide dismutase 1, p62, SQSTM1

## Abstract

Amyotrophic lateral sclerosis (ALS) is a debilitating and fatal paralytic disorder associated with motor neuron death. Mutant superoxide dismutase 1 (SOD1) misfolding and aggregation have been linked to familial ALS, with the accumulation of abnormal wild-type SOD1 species being also observed in postmortem tissue of sporadic ALS cases. Both wild-type and mutated SOD1 are reported to contribute to motoneuron cell death. The autophagic pathway has been shown to be dysregulated in ALS. Recent evidence suggests a dual time-dependent role of autophagy in the progression of the disease. PACER, also called RUBCNL (Rubicon-like), is an enhancer of autophagy and has been found diminished in its levels during ALS pathology in mice and humans. Pacer loss of function disturbs the autophagy process and leads to the accumulation of SOD1 aggregates, as well as sensitizes neurons to death. Therefore, here we investigated if constitutive overexpression of PACER in neurons since early development is beneficial in an in vivo model of ALS. We generated a transgenic mouse model overexpressing human PACER in neurons, which then was crossbred with the mutant SOD1^G93A^ ALS mouse model. Unexpectedly, PACER/SOD1^G93A^ double transgenic mice exhibited an earlier disease onset and shorter lifespan than did littermate SOD1^G93A^ mice. The overexpression of PACER in neurons in vivo and in vitro increased the accumulation of SOD1 aggregates, possibly due to impaired autophagy. These results suggest that similar to *Pacer* loss-of function, Pacer gain-of function is detrimental to autophagy, increases SOD1 aggregation and worsens ALS pathogenesis. In a wider context, our results indicate the requirement to maintain a fine balance of PACER protein levels to sustain proteostasis.

**Supplementary Information:**

The online version contains supplementary material available at 10.1186/s40659-024-00567-1.

## Background

Amyotrophic lateral sclerosis (ALS) is the most common paralytic disorder in adults with no known cure or effective treatment [1, 2]. Mutations in the gene coding for superoxide dismutase 1 (SOD1) have been associated with both familiar and sporadic cases of ALS [3, 4]. One of the commonly used animal models for ALS is the SOD1^G93A^ mouse model, which expresses the human SOD1 gene carrying the G93A mutation and presents the characteristic ALS pathology, including axonal degeneration, selective loss of motor neurons, muscle denervation, and paralysis and death [5–9]. The pathological mechanisms of ALS in motor neurons include mitochondrial malfunction, oxidative stress, autoimmunity, defects in RNA processing, the presence of toxic protein aggregates, and alterations in the mechanisms of proteostasis, the latter including autophagy [10–13]. Macroautophagy, hereafter referred to as autophagy, is an evolutionarily conserved signaling pathway that plays an essential role in cellular homeostasis due to its function in maintaining the proteostatic and metabolic balance [14]. Autophagy is a process mediated by a large collection of proteins that mediate the formation of vesicles, called autophagosomes, which may contain damaged organelles, protein aggregates, and long-lasting cytosolic proteins, destined for degradation at lysosomes [15, 16]. Notably, neurodegenerative diseases such as Parkinson’s disease, Alzheimer’s disease, Huntington’s disease, and ALS present mutations in genes related to the endosomal pathway, impairing/dysregulating vesicle trafficking and autophagy [17, 18]. Several pharmacological and genetic approaches have been used to modulate autophagy, showing that the pathway can have protective and pathogenic functions depending on the cell type analyzed and the stage of the disease [17].

We have recently identified a new gene associated with ALS, called PACER (also called C13orf18, KIAA0226l or Rubicon-like/RUBCNL) [19], which positively regulates autophagosome maturation through interaction with the UVRAG complex and antagonizes RUBICON in stimulating activity of the VPS34 kinase [19, 20]. Moreover, we and others also found PACER to be part of a complex with BECLIN1, a protein that plays an essential role in the initial phases of autophagy, a signaling pathway affected in ALS [19–22]. Previously, we reported that PACER protein levels are decreased in the lumbar spinal cord of sporadic ALS patients [19], as well as in the lumbar spinal cord of two ALS mouse models (TDP43^A315T^ and SOD1^G93A^) [19]. We also found that the loss of PACER resulted in increased accumulation of SOD1 aggregates in vitro due to impaired autophagy. By reconstituting PACER levels SOD1 aggregation could be abolished [19]. Hence, we hypothesized that the overexpression of PACER in vivo could be similarly beneficial and possibly prevent or delay disease in the SOD1^G93A^ mouse model.

Here we report the generation of a mouse model that overexpresses human PACER in neurons in the central nervous system, including the spinal cord (PACER-Tg), and their crossbreeding with the SOD1^G93A^ ALS mouse model (SOD1^G93A^-Tg). We monitored ALS-linked symptoms throughout the lifetime of those mice. We used cellular and biochemical assays in vivo and in vitro to investigate the effects of PACER overexpression on SOD1 aggregation.

## Methods

### Animals

Transgenic mice overexpressing human PACER were generated using a custom-made plasmid containing human C-terminally V5-tagged PACER (PACER-V5) under the control of the HB9 promotor [23]. PACER-transgenic mice (PACER-Tg) were generated at the Centro de Estudios Científicos (CECs), in Valdivia, Chile, using the pronuclear microinjection technique, which consists of five basic steps: (i) purification of the transgenic construction, (ii) collection of donor zygotes, (iii) microinjection of the transgenic construction in C57BL/6:CBA zygotes in a 50:50 genetic background, (iv) microinjected zygotes were transferred in pseudo-pregnant receptor mice, and (v) genotyping of pups to identify transgenic mice and analysis of transgenic expression in colony founder mice. After 7 injection attempts only three PACER-Tg founder mouse lines could be obtained. However, two lines were unable to reproduce and were lost. Hence only one line remained to be tested. The background of the remaining PACER-Tg mouse line was purified by breeding with C57BL/6 mice for four generations. Hemizygous PACER-Tg females were then bred with hemizygous SOD1^G93A^-Tg males (B6SJL-Tg(SOD1-G93A)1Gur/J obtained from The Jackson Laboratory Cat. No. 002726). Four groups of animals were obtained: non-transgenic (non-Tg), PACER-V5-Tg, SOD1^G93A^–Tg, and PACER/SOD1^G93A^–Tg. Mice were genotyped by PCR using genomic DNA extracted from tail tissue. Primers were as follows; *PACER* (human): *PACER*_R 5ʹ-GGGAGAGGCAGCATCTGTC-3ʹ, *PACER*_F 5ʹ-ATGGTGTCACAATCTACAGTCAGG-3ʹ; *Pacer* (mouse): *Pacer*_F 5ʹ-TTCACCCACCAATCAAGAGGGACA-3ʹ, *Pacer*_R 5ʹ-ACAAGACTCTGCAGATGAGTGGCA-3ʹ; h/m*Pacer* (human and mouse): h/m*Pacer*_F 5ʹ-ACACTGACCATCCTCCTTGC-3ʹ, h/m*Pacer*_R 5ʹ-GTTGTCTCTGCCAGGGAGTC-3ʹ; β-*Actin*: b*Actin*_F 5ʹ-AAGATCATTGCTCCTCCTGA-3ʹ, β-*Actin*: b*Actin*_R 5ʹ-TACTCCTGCTTGCTGATCCA-3ʹ; SOD1 (human): SOD1_F 5ʹ-CATCAGCCCTAATCCATCTGA-3ʹ SOD1_R 5ʹ-TCTTAGAAACCGCGACTAACAATC-3ʹ. The mice were housed separately under standardized conditions (room temperature 21 °C, relative humidity 55%, 12 h light/dark cycle) with free access to food and water. All animal procedures described in this study were approved by the Animal Welfare and Ethics Committee of Universidad Mayor (Protocol No. 9/2015 and Protocol No. 2/2020).

### Disease onset and progression analyses

All four groups of mice were phenotypically characterized by assessing weight loss, clinical symptoms, disease onset, and survival. The weight was consistently measured a minimum of two times per week starting at 30 days of age. To assess visual clinical symptoms, we evaluated 5 parameters starting at around 80 days of age onward a minimum of two times per week: hindlimb clasping, “electricity”, kyphosis, grooming and paralysis. Each parameter was evaluated and given a score of 0, 1, 3 or 5 according to its progression as described previously [24]. The sum of the scores was used to determine the onset and endpoint of the disease. Onset was considered when mice reached a minimum score of 7, and the endpoint was established when mice reached a score of 18 or more.

### Behavioral tests

Motor coordination and skill were assessed with the rotarod test using a rotarod apparatus (Panlab, Harvard Apparatus) [25, 26]. 4–6 weeks old mice, were trained daily 1 week before data was recorded. Training consisted of placing the mice onto the cylinder rotating at 4 rpm until animals were able to remain on it for 120 s. Once the training was complete, mice were tested twice a week by placing them onto the cylinder rotating at 4 rpm. Then rotation was continuously accelerated to 40 rpm within 2 min. Each mouse was given 3 trials to remain on the cylinder for a maximum of 120 s per trial. The latency to fall for each trial was recorded and the average was calculated.

Muscle coordination and endurance were assessed with the previously descibed hanging-wire test [27]. Briefly, a 43 cm long and 2 mm thick metallic wire was secured to two vertical stands maintained at 40 cm from the base. A timer was started as soon as the mouse was suspended in the middle of the wire. The performance of the mouse was classified with values from 1 to 5, with 1 being if the mouse fell before 10 s, 2 if the mouse held on to the wire using only its forelimbs for a period of time from 10 to 30 s; 3 if the mouse held on to the wire using its forelimbs and at least one of its hindlimbs; 4 if the mouse held on to the wire using its four limbs and the tail and; 5 if the mouse managed to descend to the base within 30 s. The position of the mouse at 30 s time was considered to assign the score. Each mouse was given 3 trials, the score for each trial was recorded, and an average score was calculated.

Exploratory behavior and anxiety-like behavior were evaluated at presymptomatic age postnatal day 90 (P90) using the open field test [28, 29]. The movement of mice was captured by a camera and analyzed using computer software. Different aspects of the behavior were measured, such as total distance traveled (m), mean speed (m/s), number of entries to the center, corner or periphery, distance traveled in the center or periphery (m) and time spent in the center, corners, or periphery (s). The open field arena consisted of four walls and floor made of white acrylic and the dimensions were 40 cm (L) × 40 cm (W) × 40 cm (H). The central area was defined as the middle 20 cm × 20 cm of the arena. The arena was illuminated at 20 lx. The arena was cleaned prior to testing each mouse using 30% ethanol. Each mouse was removed from its home cage by the tail, placed in the center of the arena, and allowed to explore for 5 min. Data were collected and analyzed using the ANY-maze software (Stoelting CO, USA).

### Cell culture and transfection

We used the NSC34 (Neuroblastoma-Spinal Cord 34) cell line [30], as a motor neuron-like model for autophagy experiments, subcellular localization studies, and mutant SOD1 aggregation. NSC34 cells were grown in Dulbecco’s modified Eagle’s medium (DMEM), supplemented with 10% of fetal bovine serum (FBS, Gibco) and 1% penicillin/streptomycin (Biological Industries, DW1012) in a 5% CO_2_ incubator at 37 °C. All transfections were performed using the Effectene reagent (Qiagen, 471 301427) according to the manufacturer’s recommendations.

### Plasmid constructs

Plasmids for mouse V5-tagged Pacer and human V5-tagged PACER have been described previously [19, 31]. Furthermore, EGFP-tagged human SOD1^WT^ and EGFP-tagged human SOD1^G93A^ or SOD1^G85R^, as well as a plasmid for EGFP alone, have been described previously [19]. Plasmid DNAs were prepared using the Qiagen plasmid midi kit (Qiagen, 12143) or the Axygen miniprep kit (Axygen, AP-MN-P-250) according to the manufacturer’s instructions.

### Autophagy analysis

NSC34 cells were transfected with vectors encoding human V5-tagged PACER or empty vector (pcDNA3.1/V5) (Invitrogen). To induce autophagy and observe autophagy flux cells were washed once with PBS and maintained with EBSS (Gibco) medium for 0.5, 2, or 4 h, similar as described in [19], and following the latest autophagy guidelines [32]. To inhibit autophagosome/lysosome fusion, cells were treated with a mix of lysosome inhibitors bafilomycin A1 (0.5 µM), an inhibitor of the lysosomal proton pumps, and protease inhibitors pepstatin (10 µg/ml) and E64D (10 µg/ml) for 4 h, all purchased from Merck.

### Lumbar spinal cord histology analysis

Tissue samples from the spinal cord of 110-day-old SOD1^G93A^ and PACER/SOD1^G93A^ mice, and non-Tg and Pacer-Tg control mice were obtained for histology and Western blot. Mice were deeply anesthetized with isoflurane and perfused transcardially with PBS followed by 4% paraformaldehyde in PBS. Hydraulic extrusion was performed to dissect the spinal cord. Once removed, spinal cords were post-fixed in 4% paraformaldehyde in PBS for 24 h at 4 °C. Afterwards, tissues were dehydrated in a sucrose gradient (7.5–15–30%) in PBS for 1 h at RT. Using the sciatic nerve as a reference, the L5 segment was sectioned with a blade followed by a second sectioning 5 mm rostral-ward the first section in order to obtain the L5 to L2 region. Dehydrated spinal cord sections were embedded in O.C.T compound (Sakura FineTek) and cryosectioned (Leica CM 1510S cryostat) at 25 μm per section.

To detect PACER protein in spinal cord sections immunofluorescence with anti-PACER antibody (Sigma Aldrich, HPA026614) was performed. Free floating spinal cord sections were exposed to antigen retrieval with citrate buffer 10 mM, pH 6 in a heat steamer for 10 min. three PBS washes, tissue sections were permealized in 0.3% Triton X-100 in PBS for 15 minutes at RT and then blocked with 3% BSA diluted in 0.05% Triton X-100 in PBS (Blocking buffer) for 2 h at RT. Afterwards, anti-PACER antibody was diluted 1:200 in blocking buffer and sections were incubated overnight at 4 °C with gentle agitation. Sections were then washed in PBS three times for 5 min each time and incubated with 1:1000 anti-rabbit Alexa-488 conjugated secondary antibody (Molecular Probes) and 1:5000 Hoechst 33342 (Molecular Probes) for nuclear staining, in blocking buffer for 2 h at RT. After three washes in PBS, sections were mounted on positive charged slides (Jiangsu Huida Medical Instrument Co., Ltd) and covered with coverslips (VWR International) using Fluoromount-G (Thermo Fisher Scientific) as mounting medium. Confocal microscopy (Leica Microsystems Leica SP8) was used to obtain microphotographs.

### Antibodies

Antibody and dilutions used were: rabbit anti-human PACER (Sigma, HPA026614) 1:1000, rabbit anti-Rubicon (Cell Signaling, 8465) 1:1000, rabbit anti-LC3B (Cell Signaling Technology, 2575) 1:1000, mouse anti-SQSTM1/p62 (Abcam, ab56416) 1:10,000, mouse anti-V5 (Thermo Fisher Scientific, R960-CUS) 1:4000, rabbit anti-Beclin1 (Santa Cruz Biotechnology, sc-11427) 1:1000, mouse anti-GFP (Santa Cruz 531 Biotechnology, sc-9996) and 1:2000, sheep anti-SOD1 (Calbiochem, 574597) 1:1000. Rabbit anti-HSP90 (Santa Cruz Biotechnologies, sc-7947) or rabbit anti-β-Actin (Cell Signaling Technology, 4967) were used as loading controls, 1:3000 or 1:1000, respectively. Secondary HRP-conjugated anti-rabbit, anti-mouse (both from Life Technologies) or anti-sheep (Sigma-Aldrich, A3415) antibodies were employed at a 1:3000 dilution.

### Western blot analysis

Cells and tissues were homogenized in Triton buffer (1% Triton in PBS 1X) containing protease inhibitor cocktail 1X by sonication. The protein concentration was determined by using the colorimetric BCA (bicinchoninic acid) assay (Pierce) according to manufacturer recommendations. Briefly, the protein quantification is based on the formation of a purple-colored complex after adding the working solution to the samples in a 96-well plate, which is measured at 562 nm. Moreover, 40 μg of protein was loaded, and the SDS-PAGE was run at 70–100 V. Western blotting was performed in a wet system from Bio-rad in PVDF membrane at 100 V for 2 h on ice. The membranes were blocked with 5% BSA in PBS 1X. The respective primary antibodies were incubated overnight at 4 °C or for 2 h at RT under agitation. Furthermore, membranes were washed 3 times for 5 min with washing solution (1xPBS/Tween 0.01%) and incubated with secondary antibody at RT for 1 h. Pierce ECL western blotting substrate was used for development according to manufacturer’s instructions. A Chemidoc equipment from Bio-rad was used for the photo-documentation of the membranes.

### Analysis of SOD1 aggregation

NSC34 cells were transiently transfected with the SOD1 expression constructs (SOD1^WT^ and SOD1^G93A^) fused to EGFP. To determine the SOD1 aggregate formation, we employed two assays: (i) Insolubility in non-denaturing detergents of SOD1 species by Western blot analysis. After 48 h of transfection, total cell extracts were prepared in 1% Triton buffer in PBS, according to the Western blot protocol described above. The samples were treated with or without reducing agent 100 mM dithiothreitol (DTT). (ii) SOD1 aggregate formation was verified by filter trap assay [19, 33]. Briefly, 1 μg/µl protein NSC34 cell extracts treated with or without DTT were filtrated through a 0.2 μm cellulose acetate membrane by using a BRL dot-blot filtration unit. SOD1 aggregates were detected with anti-SOD1 antibody, following the immunological protocol described above.

### Statistical analysis

The data are represented as the mean with its standard error (SE) in all graphs using the GraphPad Prism 9 software for statistical analysis. The Mann–Whitney U-test was used to compare two groups after the Shapiro–Wilk normality test. For survival analysis, the Log-ranked Mantel-Cox test was used. The one-way ANOVA test was used to compare more than two groups. A two-way ANOVA test was used to compare more than two variables, and a Bonferroni post-hoc test. *P* values less than 0.05 were considered significant.

## Results

### PACER transgenic mice show high expression of human PACER in the cortex, hippocampus, and spinal cord

**Fig. 1:**
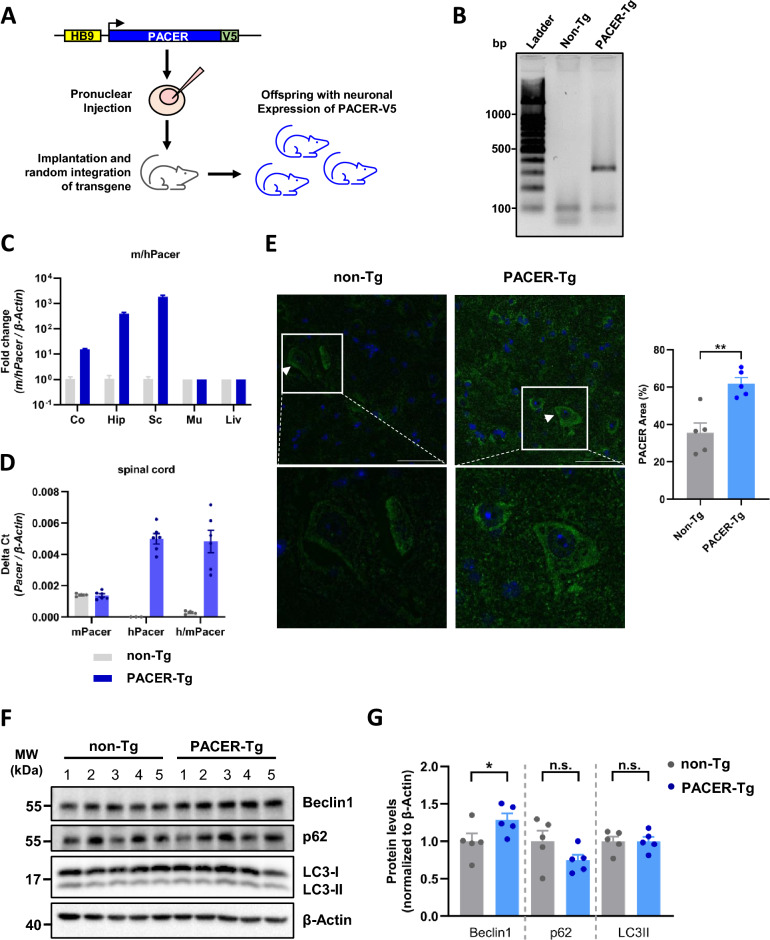
Generation of transgenic mice expressing V5-tagged PACER in neurons. **A** Graphical scheme of microinjection strategy. **B** Genotyping of PACER transgenic mice. PCR detection of HB9-PACER-V5 transgene (single band, 334 bp) in heterozygous PACER-Tg mice. Wild-type mice do not present amplified products for these primers. **C**, **D** mRNA levels were determined by qPCR in PACER-Tg (n = 7) versus non-Tg mice (n = 7). **C** Primers detecting mouse *Pacer* and human *PACER* with equal specificity were used to detect total *Pacer/PACER* mRNA levels in cortex, hippocampus, spinal cord, muscle, or liver. Fold change of mRNA levels was calculated using *β-Actin* mRNA levels as a reference. **D** Primers detecting specifically mouse *Pacer* mRNA or human *PACER* mRNA or both with equal specificity were used to determine expression levels of endogenous versus exogenous *PACER* mRNA. *β-Actin* mRNA levels were determined as a reference. **E** Detection of PACER in motor neurons of the lumbar spinal cord of PACER-Tg mice by immunofluorescence and confocal microscopy. **F** Western blot detection of autophagy proteins Beclin1, p62 and LC3-II in the lumbar spinal cord of PACER-Tg (n = 5) versus non-Tg (n = 5) mice. β-Actin protein levels were used as a loading control. **G** Quantification of protein levels of Beclin1, p62 and LC3-II relative to β-Actin detected in **F**.  In **E** and **F** statistical analysis using Mann–Whitney U-test was performed. Data is presented as means ± S.E.M. p value: p > 0.05: n.s., non-significant, *p < 0.05; **p < 0.01

Previously, we reported on the loss of Pacer protein in motor neurons during ALS pathogenesis in the SOD1^G93A^ mouse model [19]. Hence, we aimed to generate a transgenic mouse model that overexpressed human PACER in motor neurons. A plasmid construct was designed that carries an expression-cassette containing C-terminally V5-tagged human PACER protein under the control of the HB9 promotor (Fig. 1A). The HB9 promotor was selected for its known expression in motor neurons [29]. From 7 rounds of pronuclear injections only 3 pups positive for the construct integration could be obtained, from those three PACER-Tg founders only one PACER-Tg mouse line could be established, since 2 of 3 founders were unable to reproduce. To date, the PACER-Tg line has been maintained for more than 10 generations in the C57BL/6 background and retains transgene expression as confirmed by routine genotyping PCR (Fig. 1B). Expression of the PACER transgene was assessed by real-time quantitative PCR using primers that recognize mouse *Pacer* and human *PACER* mRNA sequences, with equal efficiency. As expected, high expression of human *PACER* in PACER-Tg mice compared to endogenous mouse *Pacer* in non-Tg mice was observed in neuronal tissues, cortex, hippocampus, and spinal cord, where the overall highest was in the spinal cord (Fig. 1C). In non-neuronal tissues, such as muscle and liver, no PACER overexpression was observed (Fig. 1C). Using three sets of primers that recognize either human, mouse or both mRNA sequences of *Pacer/PACER* we determined the level of *PACER* overexpression specifically in the spinal cord (Fig. 1D). Furthermore, with immunofluorescence (IF) assays we determined the presence of PACER transgene in motor neurons of the spinal cord ventral horn of PACER-Tg mice (Fig. 1E). Since PACER is known to be a regulator of autophagic capacity, we also assessed the levels of key proteins involved in the process in spinal cord samples by Western blot. We found that protein levels of Beclin1 were increased, while protein levels of p62 and LC3-II were not significantly affected (Fig. 1F and G). Thus, viable and stable PACER transgenic mice could be generated, which overexpress human PACER in neurons of the spinal cord and brain.

### ***Overexpression of PACER in neurons accelerates disease onset, worsens symptoms, and shortens the lifespan of SOD1***^***G93A***^***-Tg mice***

**Fig. 2:**
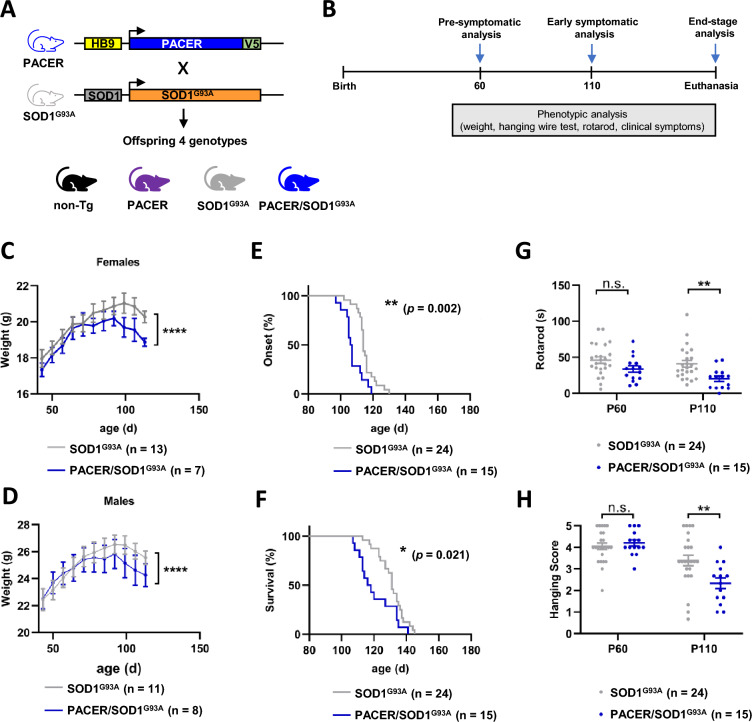
Phenotypic analysis across the lifespan of SOD1^G93A^ versus PACER/SOD1^G93A^-Tg mice. **A**, **B** Schematic representation of experimental setup. **A** Briefly, heterozygous SOD1^G93A^-Tg mice and heterozygous PACER-Tg mice were crossed to obtain four experimental groups, (i) non-Tg, (ii) PACER-Tg, (iii) SOD1^G93A^-Tg, and (iv) PACER/SOD1^G93A^-Tg mice. **B** Phenotypic analyses were performed of all 4 groups throughout their life, starting at day postnatal day 30 until euthanasia. **C**–**H** Phenotypic data comparing SOD1^G93A^-Tg (n = 24) versus PACER/SOD1^G93A^-Tg (n = 15) are shown. Data of control groups non-Tg (n = 15) versus PACER-Tg (n = 15) mice is shown in Supplementary Fig. 1. **C** The body weight curves of female SOD1^G93A^ (n = 13) versus female PACER/SOD1^G93A^ (n = 7), and **D** male SOD1^G93A^ (n = 11) versus male PACER/SOD1^G93A^ (n = 8) mice. **E** Onset of clinical symptoms and **F** survival was determined. **G** Rotarod and **H** hanging wire test performance was assessed at pre-symptomatic (pre-onset) time point P60 and symptomatic (post-onset) timepoint P110. The mice were monitored twice weekly from P30 to P130. In **C**–**H** statistical analysis using (**C**) and (**D**) two-way ANOVA with Bonferroni post-hoc test, **E** and **F** Log-ranked Mantel-Cox, **G** and **H** Mann–Whitney U-test. Data is presented as means ± S.E.M. p values: p > 0.05: n.s., non-significant; *, p < 0.05; **, p < 0.01; ***, p < 0.001; ****, p < 0.0001

To investigate the effect of PACER overexpression on the ALS phenotype observed in SOD1^G93A^-Tg mice, we crossbred PACER-Tg mice with SOD1^G93A^ -Tg mice to generate PACER/SOD1^G93A^-Tg mice (Fig. 2A). We then assessed pathological parameters throughout life (Fig. 2B). Body weights of female and male PACER/SOD1^G93A^ mice were significantly declined after 100 days of age compared to female and male SOD1^G93A^ mice, respectively (Fig. 2C). The body weight of males and females of the control groups non-Tg and PACER-Tg was comparable and continuously increased throughout the study period (Supplementary Fig. 1A). These results indicate that the neuronal overexpression of PACER in SOD1^G93A^ mice accelerates disease associated body weight loss, a critical signal of ALS phenotype in preclinical models. To assess the effect of PACER overexpression on the onset and progression of disease in SOD1^G93A^ mice, we performed lifespan analysis comparing SOD1^G93A^ and PACER/SOD1^G93A^ mice. We did not observe significant differences between genders; hence we combined data from both genders for analysis. Furthermore, the clinical disease onset of PACER/SOD1^G93A^ mice (105 days) was significantly earlier than that of SOD1^G93A^ mice (114 days) (Fig. 2D), while the mean lifespan of PACER/SOD1^G93A^ mice (113 days) was significantly shorter than that of SOD1^G93A^ mice (130 days) (Fig. 2E). Motor function was assessed with rotarod and hanging wire test as previously described [27]. We compared the performances of SOD1^G93A^-Tg versus PACER/SOD1^G93A^-Tg animals at 60 days (pre-onset) and 110 days (post-onset) of age. At 60 days of age both groups performed comparable in rotarod and hanging wire test (Fig. 2F and G), while at 110 days the performance of both groups declined compared to pre-onset. Nonetheless, the PACER/SOD1^G93A^ mice performed significantly worse than the SOD1^G93A^ mice in both rotarod and hanging wire test (Fig. 2F and G). The performance in rotarod and hanging wire test of the control groups non-Tg and PACER-Tg was comparable to each other at both P60 and P110, indicating no significant decline of performance at P110 compared to P60 (Supplementary Fig. 1B and 1C). Autophagy activity has been associated with behavior and cognitive parameters in mice [34–36]. The behavior of the non-Tg mice presents a characteristic normal exploratory impulse, where they homogeneously moved throughout the field, unlike the SOD1^G93A^-Tg mice, which tend to have higher levels of anxiety when exposed to open fields and therefore tend to stay mainly in the periphery (Supplementary Fig. 2A and B), which has been previously observed [37]. The PACER-Tg mice behaved like the non-Tg mice with no significant abnormalities, while the PACER/SOD1^G93A^-Tg mice behaved similar to the SOD1^G93A^-Tg mice, displaying a comparably anxious phenotype (Supplementary Fig. 2B). No significant difference was observed between the level of anxiety of the PACER/SOD1^G93A^ versus the SOD1^G93A^-Tg mice.

In summary, these results indicate that neuronal PACER transgene overexpression in SOD1^G93A^ mice significantly accelerates disease onset and decreases average lifespan, as well as significantly worsens motor function while having no effect on anxiety behavior.

### ***Neuronal overexpression of PACER results in increased misfolded SOD1 levels in the spinal cord of symptomatic SOD1***^***G93A***^*** mice***

**Fig. 3:**
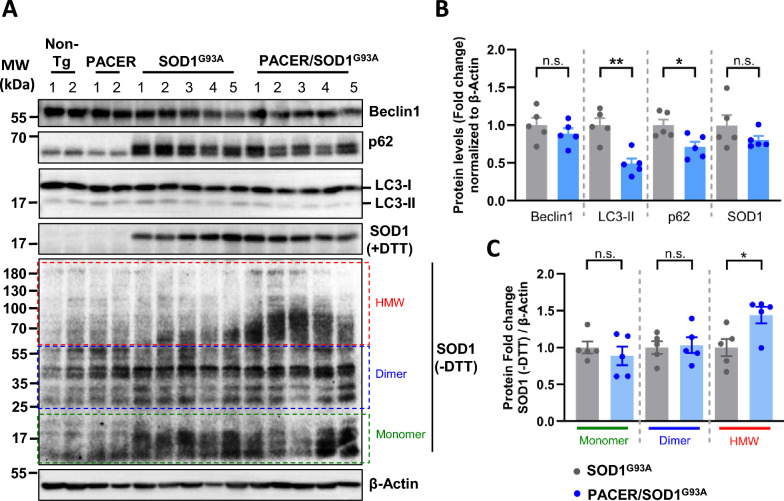
Exaggerated autophagy dysfunction increases SOD1 aggregate accumulation in PACER/SOD1^G93A^ mice. **A**–**C** Levels of autophagy-related proteins and SOD1 aggregates were determined in the lumbar spinal cord of symptomatic (P121) SOD1^G93A^-Tg (n = 5) and PACER/SOD1^G93A^-Tg (n = 5) mice. Levels of the same proteins in non-Tg (n = 2) and PACER-Tg (n = 2) serve as references. **A** Western blot analysis of autophagy markers Beclin1, p62 and LC3-II, as well as SOD1 under reduced (+DTT) and non-reduced (−DTT) conditions to detect SOD1 aggregates. β-Actin was detected as a loading control. **B** Quantification of the fold change of autophagy protein levels as well as total SOD1 (+DTT) in the spinal cord, normalized to β-Actin levels, are shown. **C** Quantification of SOD1 high molecular weight (HMW) aggregates, as well as Dimers and Monomers under non-reducing (−DTT) conditions was performed using β-Actin levels as a loading control. In **B** and **C** statistical analysis using Mann–Whitney U-test was performed. Data is presented as means ± S.E.M. p value: p > 0.05: n.s., non-significant, *p < 0.05; **, p < 0.01

We and other reported that Pacer functions as an autophagy enhancer and thus can affect the levels of SOD1 aggregation [19, 20]. Hence, to assess the effect of PACER overexpression on autophagy and SOD1 aggregation in vivo, we determined the protein levels of autophagy markers LC3-II, p62 and Beclin1 in the lumbar spinal cord tissue of symptomatic (P121) SOD1^G93A^ and PACER/SOD1^G93A^ mice by Western blot (Fig. 3A and B). As expected, levels of LC3-II and p62 are accumulated in SOD1^G93A^ mice, indicating an impairment in autophagy [19, 38]. The protein levels of p62 and LC3-II were decreased in PACER/SOD1^G93A^ mice compared to SOD1^G93A^ mice, while the levels of Beclin1 remained unaffected (Fig. 3A and B), but not at the basal wild-type levels. To investigate if PACER overexpression in SOD1^G93A^ mice affects the accumulation of SOD1, we performed a Western blot of lumbar spinal cord samples under reduced (+DTT, to detect total SOD1 levels) and non-reduced (−DTT, to detect SOD1 aggregates) conditions (Fig. 3A and C). Increased SOD1 high molecular weight (HMW) aggregation was found in PACER/SOD1^G93A^ mice compared to SOD1^G93A^ mice (Fig. 3A, SOD1 −DTT panel). These results indicate that PACER overexpression appears to exaggerate mutant SOD1 misfolding leading to the accumulation of SOD1 aggregates. Together, these results suggest that PACER overexpression may induce an imbalance in autophagic function, which may be related to the accumulation of SOD1 aggregates.

### Motor neuronal autophagy is impaired by PACER overexpression

**Fig. 4:**
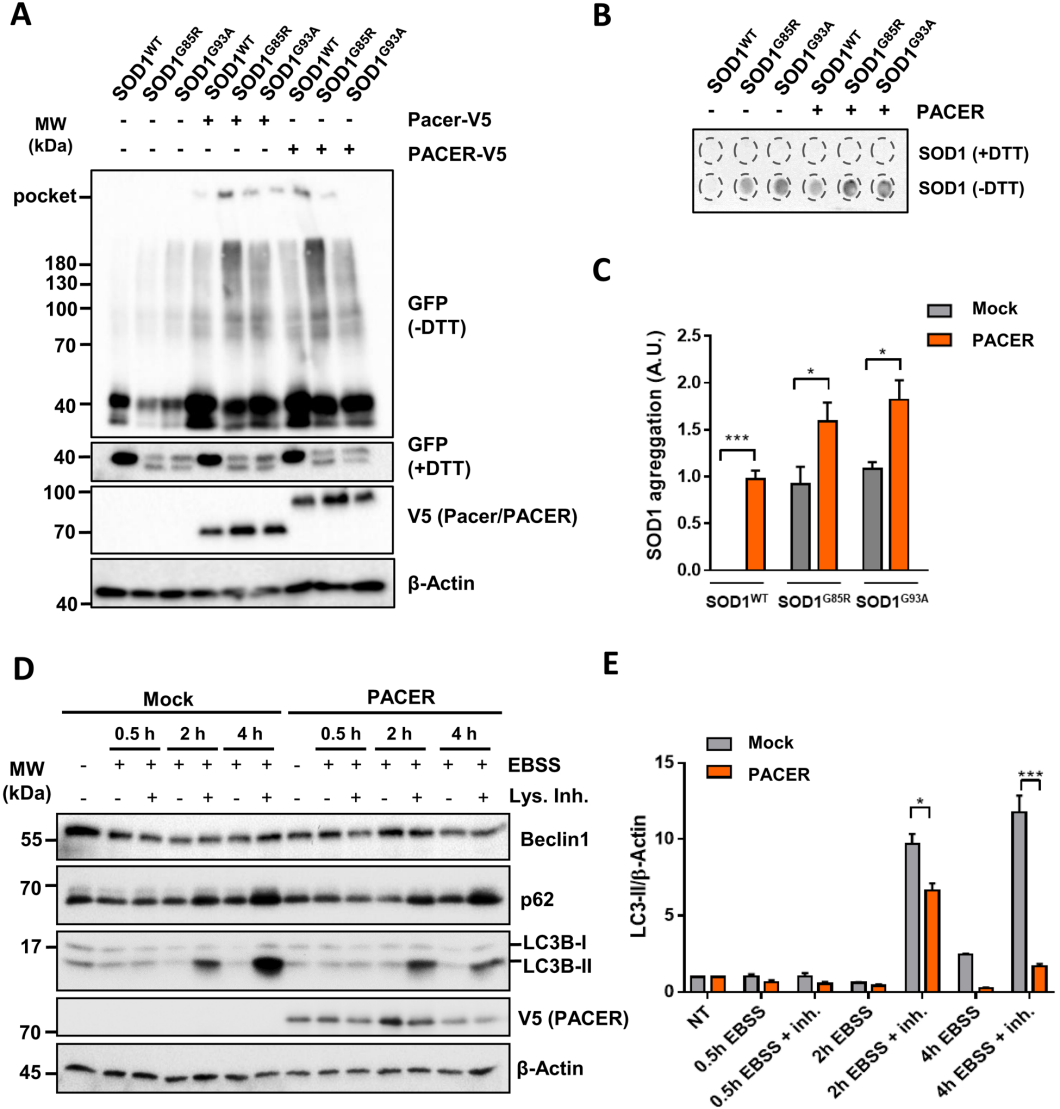
PACER overexpression in NSC34 cells increases SOD1 aggregation and impairs autophagy. **A**–**C** NSC34 cells were transiently co-transfected with expression vectors for human wild-type SOD1 (SOD1^WT^) or mutant SOD1^G85R^ or SOD1^G93A^ all fused to EGFP together with a vector for mouse or human V5-tagged Pacer or PACER, respectively, or a mock control. After 48 h, SOD1 levels under reduced (+DTT) and non-reduced (−DTT) conditions were monitored in cell extracts prepared in 1% Triton X-100 buffer by western blot. Representative blots of 3 experiments are shown. **B** Filter trap assay (n = 3), and **C** densitometric quantification of the filter trap assay of SOD1 mutants and wild-type (n = 3). Mean and SEM are shown. **D** and **E** Autophagy flux was performed in NSC34 cells transiently transfected with a plasmid for human V5-tagged PACER or plasmid vector alone (Mock) and treated with EBSS and/or lysosome inhibitors (Lys. Inh.: Bafilomycin A1, E64D, and leupeptin) at different time points (0.5 h, 2 h, 4 h). **D** Cell extracts were subjected to western blot. LC3B-II, p62, Beclin1 and V5 expression were verified. β-Actin levels were used as a loading control (n = 3). **E** Autophagy flux was quantified by densitometric analysis of LC3-II (n = 3) normalized to β-Actin. Mean and SEM are shown. In **C** and **E** statistical analysis was performed using One-way ANOVA. p values: n.s., non-significant, *, p ≤ 0.05; **, p ≤ 0.01;***, p ≤ 0.001

We then investigated if PACER overexpression in motor neuronal cell culture recapitulated the increased accumulation of SOD1 aggregates observed in vivo. We co-expressed EGFP-tagged wild-type SOD1, or the SOD1 mutants G85R and G93A together with V5-tagged human PACER (PACER-V5) or V5-tagged mouse Pacer (Pacer-V5) to investigate a possible species-specific difference in their effect. Both the overexpression of PACER-V5 and Pacer-V5 had comparable effects on SOD1^G93A^ and SOD1^G85R^, increasing their aggregation (Fig. 4A). Furthermore, we also observed increased aggregation of SOD1^WT^ when both PACER-V5 or Pacer-V5 were co-expressed (Fig. 4A). In line, with our results in Western blot we also confirmed the increased formation and accumulation of high molecular weight SOD1 aggregates (SOD1^WT^, SOD1^G85R^ and SOD1^G93A^) in filter trap when PACER-V5 is overexpressed (Fig. 4B and C). These results suggest that human PACER and mouse Pacer behave comparable, and both can cause increased SOD1 aggregate accumulation independent of and which mutation is present in SOD1. Since, not only mutant SOD1, but also SOD1^WT^ aggregates are found to be accumulating when PACER is overexpressed, and SOD1 monomer levels are equal for WT and mutant proteins, we speculated that increased amounts of PACER resulted in an unexpected impairment of the autophagy process. To determine if autophagy was affected by the overexpression of PACER-V5, we performed an autophagy flux experiment. Indeed, we found that PACER-V5 overexpression impaired autophagic flux significantly, showing a decreased LC3B-dependent autophagosome formation, observed by a reduced LC3II accumulation under lysosome inhibition compared to control (Fig. 4D and E). Together, these results support our results in vivo, where the overexpression of PACER impairs autophagy and results in the accumulation of SOD1 aggregates.

## Discussion

ALS symptoms are associated with the selective dysfunction and eventual death of motor neurons in the motor cortex and spinal cord responsible for voluntary muscular contractions, resulting in progressive paralysis [2]. The homeostasis of postmitotic cells, such as neurons, depends on their high capacity for basal autophagy, which is responsible for the clearance of deficient organelles or protein aggregates. We have previously described that PACER is associated with ALS pathology, finding that its protein levels are significantly decreased both in the spinal cord from ALS patients, as well as murine models of ALS [19]. The relationship between PACER levels and motor neuron survival was further validated in cell culture studies, showing that PACER is an enhancer of autophagic capacity, and PACER loss of function impairs autophagy favoring the development of the pathophysiology of ALS [19]. Mechanistically, PACER functions in tandem with key components of the autophagy machinery, such as BECLIN1 [22], to drive the PI3KC3/VPS34 kinase activity, which is antagonized by RUBICON [20, 22]. PACER was reported also to mediate the fusion of the autophagosome and lysosome vesicles by binding to the autophagosome SNARE STX17 [21]. The autophagy enhancer activity of Pacer was also explored in treating inflammatory diseases such as inflammatory bowel disease using cell therapy with mesenchymal stem cells (MSCs) [31]. We reported that in MSCs, Pacer is upregulated in response to pro-inflammatory stimuli like TNFα. Promoting its overexpression in MSCs increases their autophagic capacity and their ability to suppress harmful immune responses in the context of cellular therapy [31].

In the central nervous system, Pacer was found localized mainly to neurons, including motor neurons of the spinal cord of the murine SOD1^G93A^ ALS mouse model [19]. The levels of Pacer protein are significantly decreased in motor neurons in the late phases (symptomatic) of ALS disease when SOD1^G93A^ mice lose the ability to clean themselves or to walk normally [19]. Moreover, in our previous in vitro studies using the motor neuron cell line NSC34 we observed that the repression of Pacer by knockdown generates a significant increase in SOD1 aggregates which was associated with a disturbance of autophagic flux [19]. Furthermore, under Pacer loss of function, motor neuron-like cells were found to be more vulnerable to cell death caused by SOD1 mutant expression [19]. However, the reconstitution of Pacer-depleted cells with human PACER decreased SOD1 aggregation levels and rescued their survival under SOD1^G93A^ expression [19]. These findings suggested a protective role of PACER from neurodegeneration or death of motor neurons in vivo. As the logical next step, in this study, we aimed to investigate if the constitutive overexpression of human PACER in neurons since early development could have a positive effect on disease onset and/or progression in SOD1^G93A^ mice as well as disease symptoms. Contrary to our expectations, constitutive overexpression of human PACER in neurons led to an earlier onset of symptoms (both clinical and motor) as well as a reduction in lifespan, worsening the overall outcome. PACER-transgenic (PACER-Tg) mice showed no obvious phenotype over several generations compared to non-transgenic (non-Tg) control mice. However, when PACER-Tg were crossed with SOD1^G93A^-transgenic (SOD1^G93A^-Tg) mice, the resulting PACER/SOD1^G93A^-Tg mice showed unexpectedly exacerbated weight loss, accelerated disease onset and motor deterioration, as well as reduced average lifespan compared to SOD1^G93A^-Tg littermates.

Furthermore, our results show that PACER overexpression, both in vivo and in vitro, impairs autophagic function, resulting in an increase of SOD1 protein aggregate accumulation. Given that PACER is an enhancer of autophagy, we expected that the gain-of function of PACER at the neuronal level should favor the clearance of polyubiquitinated proteins via autophagy, in addition to the cytotoxic protein aggregates of SOD1 that have not been able to be degraded by the proteasome under ALS conditions. Also, previously, we reported that Pacer loss-of function generated an increase in aggregates of mutant SOD1, and even promoted the de novo aggregation of wild-type SOD1, which may be due to disruption of autophagy in the absence of Pacer [19]. Interestingly, our results with overexpression of human and mouse Pacer result in a similar increase of aggregation of both wild and mutant SOD1 as observed previously in cells lacking Pacer [19], suggesting that Pacer levels in neurons are tightly regulated to preserve neuronal homeostasis. A comparable effect has been previously observed by modulating p62 levels, where the knockout of p62 had the same effect on SOD1 aggregation as had its overexpression [39–41]. These results suggest that the levels of certain proteins involved in dynamic processes such as autophagy are highly controlled, and imbalances outside of a certain range in both directions could have detrimental consequences for the cell. PACER and p62 protein levels could be subject to a finely tuned regulatory machinery of the autophagic pathway. Similarly, the modulation of autophagy as a therapeutic target for ALS has presented great complexities and difficulties, since several studies have suggested that multiple factors have to be considered for a beneficial outcomes, such as the type of intervention, the cell type targeted, the state of the disease as well as the state of the pathway affected in this particular timepoint of pathological progression [6, 24, 39–44] and reviewed in [24, 45]. Hence, varying results have been obtained in different ALS models. One of them, using the murine model SOD1^G93A^ with conditional neuronal knockout of Atg7 to modulate autophagy in different stages of the disease, showed that autophagy in early stages plays a neuroprotective role, maintaining the neuromuscular junctions [6]. However, in later stages it promotes disease progression through non-autonomous cellular mechanisms, stimulating neuroinflammation through up-regulation of c-Jun, which triggers astrogliosis and microgliosis [6].

In our study we increase Pacer levels over the normal range already in early development due to the use of the HB9 promotor, which might result in an imbalance in the pathway or an overcapacity of autophagy when not needed. It has been found that autophagy is precisely controlled during specific stages of prenatal and postnatal development [46, 47]. Specifically in the developing central nervous system dysregulated autophagy can affect the number of neural progenitors which can affect neuronal outgrowth, connectivity, and functionality with cellular, tissue structural and behavioral consequences as the organism ages [46, 48]. Another possibility, which could explain our results, is based on the fact that the phenotype of ALS disease has been shown to be affected by nutritional conditions [49, 50]. In the murine model of ALS, mice that express the mutant version of 43 kDA of the protein TARDBP, it has been observed that high-fat foods extend their lifespan [51], a phenomenon that has also been observed in mice that express the mutant version of SOD1, when the activation of AMP-activated protein kinase (AMPK) is induced, a protein that acts as an energy sensor [52]. Despite maintaining a normal food availability, we observed an earlier decrease in the body weight of the PACER/SOD1^G93A^ mice, compared to the SOD1^G93A^ mice in both sexes. These differences begin to be observed from pre-symptomatic stages, close to 70 days, before clear clinical symptoms. Interestingly, it has been reported that the activity of PACER in the maturation of autophagosomes is regulated by the nutritional or energy balance of the cell. Under conditions rich in nutrients, PACER is phosphorylated in serine 157 by mTOR, which inhibits the association of PACER with STX17 and the HOPS complex, thus preventing the maturation of the autophagosome [53].

## Conclusion

In summary, our study further highlights the complexity of the pathophysiology of ALS required to be considered for its understanding as well as potential therapeutic treatments. Our data suggest that the timepoint of treatments could be crucial depending on the actual neuronal necessity, where a fine-tuning of dynamic pathways such as autophagy has to be taken into account for target selection. Regarding the potential therapeutic benefit of PACER function more studies are needed to determine if a reconstitution of the levels of PACER when needed rather than a constitutive overexpression is a better approach. It remains to be determined which is the best time point for such a reconstitution in vivo in the context of ALS.

## Supplementary Information

Supplementary Material 1.

## Data Availability

Data sharing is not applicable to this article as no datasets were generated or analysed during the current study.
