# Supplementary material for: Overexpression of autophagy enhancer PACER/RUBCNL in neurons accelerates disease in the SOD1G93A ALS mouse model
Source: Biol Res. 2024 Nov 17;57:86. doi: 10.1186/s40659-024-00567-1 (PMC11571584; doi:10.1186/s40659-024-00567-1)
Supplement: Supplementary file 1 — Supplementary Material 1. [file 40659_2024_567_MOESM1_ESM.pdf]

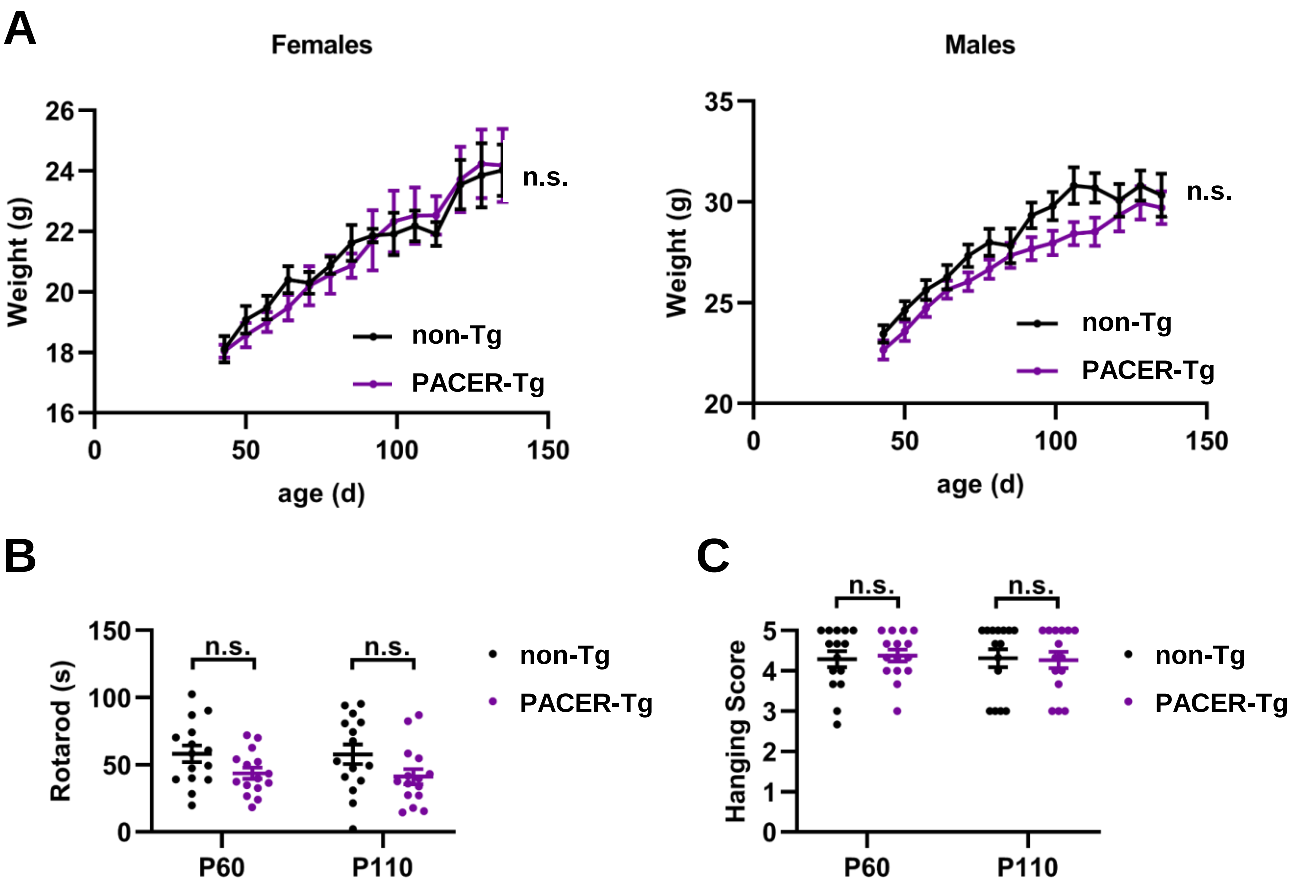

Supplementary Figure 1

Supplementary Figure 1: Phenotypic analysis across the lifespan of non-Tg versus PACER-Tg mice. (A-C) Control groups, non-Tg and heterozygous PACER-Tg mice were obtained as described in Figure 2A. Phenotypic analyses were performed of all 4 groups throughout their life, starting at day postnatal day 30 until euthanasia. Phenotypic data comparing non-Tg (n=15) versus PACER-Tg (n=15) are shown. Data of SOD1^{G93A}-Tg (n=24) versus PACER/SOD1^{G93A}-Tg (n=15) mice is shown in (Figure 2C-G). (A) The body weight curves of male non-Tg (n=10) versus male PACER-Tg (n=9) and female non-Tg (n=5) versus female PACER-Tg (n=6) mice. (B) Rotarod and (C) hanging wire performance was assessed at pre-symptomatic (pre-onset) time point P60 and symptomatic (post-onset) timepoint P110. The mice were monitored twice weekly from P30 to P130. In (A-C) statistical analysis using (A) two-way ANOVA with Bonferroni post-hoc test, (F) and (G) Mann-Whitney U-test. Data is presented as means ±S.E.M. p values: $p > 0.05$: n.s., non-significant; *, $p < 0.05$; **, $p < 0.01$; ***, $p < 0.001$; ****, $p < 0.0001$.

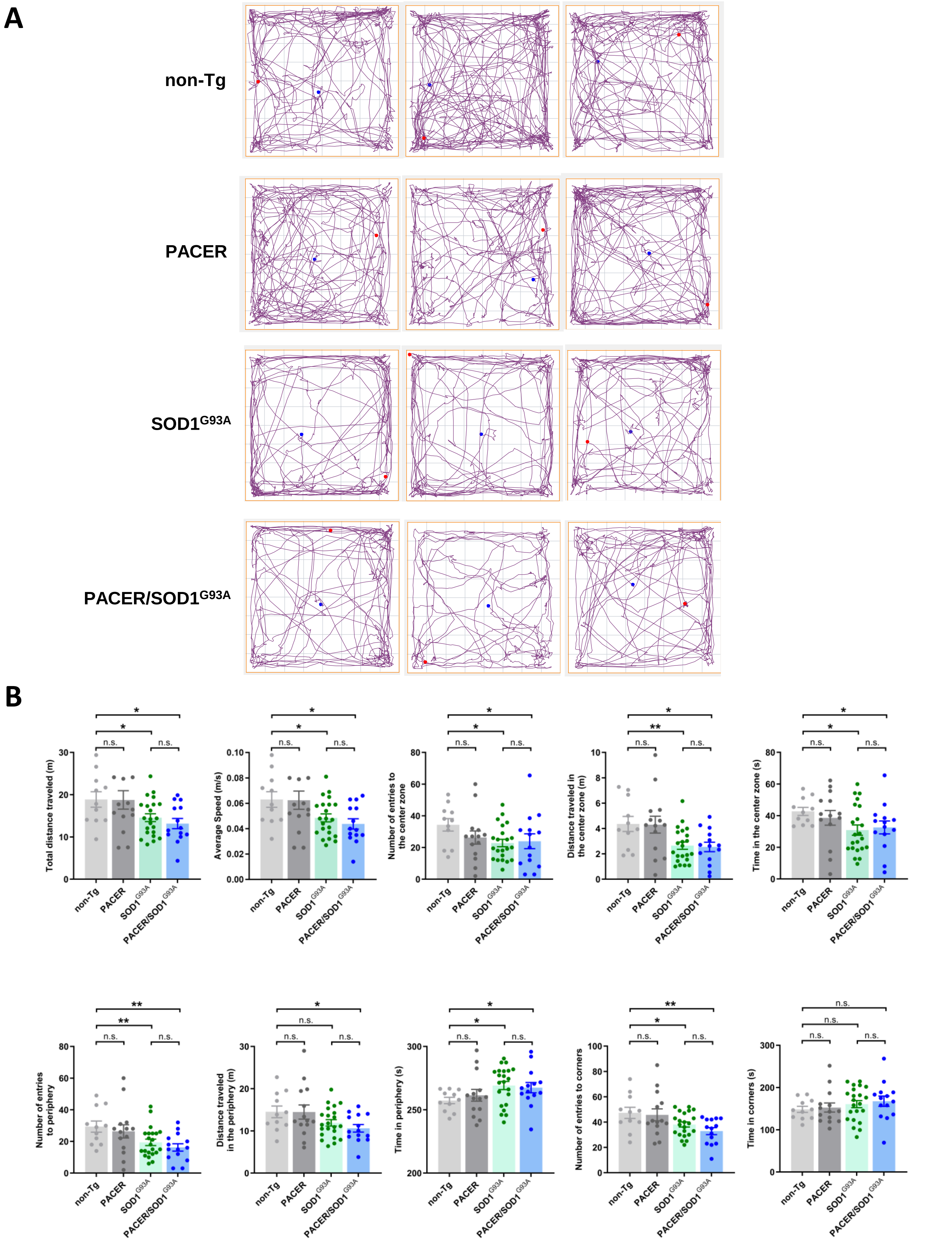

Supplementary Figure 2

Supplementary Figure 2: Open field test of pre-symptomatic SOD1^{G93A}-Tg, PACER/SOD1^{G93A}-Tg, PACER-Tg and non-Tg mice at age P90. (A) Three representative traces of the tracks travelled by three different mice of each of the four groups, (non-Tg, PACER-Tg, SOD1^{G93A}-Tg, and PACER/SOD1^{G93A}-Tg). The blue dot indicates initial position at the beginning of the track and the red dot the end of the track. The data was analyzed using video tracking software (ANY-maze, Stoelting CO, USA). **(B)** Analyses of different behavioral parameters assessed for mice of each group, non-Tg (n=11), PACER-Tg (n=14), SOD1^{G93A}-Tg (n=22), PACER/SOD1^{G93A}-Tg (n=14): total distance traveled (m), mean speed (m/s), number of entries to the center, corner or periphery, distance traveled in the center or periphery (m) and time spent in the center, corners, or periphery (s). Statistical analysis was performed using Mann-Whitney U-test. Means ± S.E.M. are shown for each group. p values: $p > 0.05$: n.s., non-significant; *, $p < 0.05$; **, $p < 0.01$.
